# High Quality of Life Reduces Depression, Hopelessness, and Suicide Ideations in Patients in Forensic Psychiatry

**DOI:** 10.3389/fpsyt.2019.01014

**Published:** 2020-01-23

**Authors:** Michael Büsselmann, Stefanie Nigel, Stefanie Otte, Maximilian Lutz, Irina Franke, Manuela Dudeck, Judith Streb

**Affiliations:** ^1^Department of Forensic Psychiatry and Psychotherapy, Ulm University, Ulm, Germany; ^2^Department of Forensic Psychiatry, Psychiatric Services Graubuenden, Cazis, Switzerland

**Keywords:** quality of life, forensic psychiatry, suicide, suicide ideations, social conditions, depression, hopelessness

## Abstract

**Background:**

Suicides are more common in forensic patients than in the general population. Two reasons for this discrepancy are discussed: (1) Suicides are the consequence of maladaptation to the restrictive living conditions in forensic psychiatry, and (2) suicides are explained by the demographic, social, and psychosocial characteristics of the inmates themselves, i.e., suicides happen because the inmates belong to a particularly vulnerable group. Therefore, the present study aimed to analyze the relationship between quality of life, as an indicator of the restrictive living conditions, and hopelessness, depression, and suicide ideations in a sample of forensic patients.

**Methods:**

We assessed quality of life with a German version of the Measuring the Quality of Prison Life questionnaire that had been adapted to forensic hospitals (MQPL-forensic) and depressive symptoms with the Beck Depression Inventory, hopelessness with the Beck Hopelessness Scale, and suicide ideations with the Beck Scale for Suicide Ideation. The study included a total of 159 patients in 12 German forensic psychiatric hospitals who had been admitted in accordance with Section 64 of the German Criminal Code. We analyzed the relationships between quality of life and depression, hopelessness, and suicide ideations on the patient and hospital levels. Hospital characteristics were generated by aggregating the MQPL-forensic variables measured at the patient level.

**Results:**

In generalized estimating equation models, the MQPL-forensic total score and almost all the subscale scores were significant negative predictors of depressive symptoms, hopelessness, and suicide ideations at the patient and hospital levels. At the patient level, patients who experienced a supportive welcome at the hospital, good relationships with their therapists, respectful interactions, transparent decisions, and supportive therapeutic approaches were significantly less depressed, less hopeless, and less likely to consider suicide. At the hospital level, good relationships with therapists and respectful interactions were significant negative predictors of these variables.

**Discussion:**

The results indicate that the social framework within forensic psychiatric hospitals influences the frequency of suicide ideation and the severity of depressive symptoms and hopelessness among forensic patients. Forensic-psychiatric hospitals should be aware of these significant relationships and try to improve patients’ quality of life.

## Introduction

According to Statista (1), 17.7 per 100,000 men die of suicide each year in Germany. The numbers are even higher when we consider people in secure facilities. Voulgaris et al. (2) report a mean suicide rate of 103 per 100,000 male prisoners and 163 per 100,000 male forensic psychiatric inpatients. These dramatically high rates require preventive action.

Three models to explain suicides in prisons are discussed in the literature: the deprivation model, importation model, and combined model (3, 4). According to the deprivation model, suicides are the consequence of maladaptation to the restrictive living conditions in prison (e.g., loss of autonomy, sense of security, freedom, and contact with family and friends) (3, 5). Maladaptation can take the form of violence, self-aggression, anxiety, depression, psychological stress, and suicide (4). According to the importation model, however, suicides in prisons are explained by the demographic, social, and psychosocial characteristics of the prisoners themselves, i.e., the majority of prisoners belong to a particularly vulnerable group and thus bring the risk of suicide from outside into prison (3, 6); this assumption is supported by the fact that suicides inside and outside prisons are subject to the same risk factors (e.g., male sex, mental illness, and high propensity to violence) (4). Both models have weaknesses. For example, the deprivation model cannot explain why one inmate commits suicide but another does not, and the importation model refers to general risk factors but does not include special prison settings. The so-called combined model tries to compensate for these weaknesses by combining both theories (4). This model assumes that because of their individual vulnerabilities prisoners adapt differently to the different living conditions in prison and that both factors (vulnerability and living conditions) interact (4).

Although many studies have investigated imported vulnerability in connection with suicidal behavior (2, 7–10), only a few have focused on quality of life in prisons (11–13). Quality of life is a complex and multidimensional construct that the WHO (14) describes as follows: “… an individual’s perception of their position in life in the context of the culture and value systems in which they live and in relation to their goals, expectations, standards and concerns. It is a broad ranging concept affected in a complex way by the person’s physical health, psychological state, personal beliefs, social relationships and their relationships to salient features of their environment.” Various methods exist to capture quality of life in prisons (15, 16). One such method, the Measuring the Quality of Prison Life [MQPL; (12, 17)] questionnaire, was developed by Liebling and colleagues on the basis of interviews with a total of 100 prisoners in seven different prisons. In close cooperation with the inmates, the working group identified several parameters that are particularly important for prisoners’ quality of life, i.e., respect, humanity, staff-prisoner relationships, support, trust, fairness, order, safety, well-being, personal development, family contact, and decency (12). In a study entitled “Revisiting prison suicide: the role of fairness and distress” (3), Liebling et al. showed how important these parameters are for the health and well-being of people in secure facilities. For this study, they interviewed 2,608 prisoners in 12 prisons in England. The results showed that prisoners’ self-assessed quality of life, as measured by the MQPL, was significantly correlated with psychological distress, as measured by the General Health Questionnaire (GHQ-12) (18). The higher the inmates rated the dimensions physical safety, respect, relationship, fairness, dignity, frustration, clarity, security, and family contact, the lower was their psychological distress. Furthermore, the study found that prisoners’ psychological distress was positively correlated with the prison suicide rate (3).

The present study investigated quality of life in forensic psychiatry. In Germany, forensic psychiatric hospitals accommodate people who have committed a serious crime because of a mental or substance use disorder. In some respects, the living conditions in forensic psychiatric hospitals are similar to those in prisons. Prisoners and forensic patients are deprived of liberty, autonomy, heterosexual relationships, and personal possessions; however, there are also differences. Because forensic patients have a mental or substance use disorder, they are cared for by doctors, psychologists, and nurses and receive treatment. The treatment objectives are to reduce the risk that the patients pose to society and facilitate their reintegration into society (19). Initial studies have been performed on quality of life in forensic psychiatry, but they mostly focused on mentally ill patients (schizophrenia and personality disorders) (20, 21) or community-based forensic psychiatric treatment (22–24). Therefore, we used the MQPL adapted for forensic psychiatry (25) (MQPL-forensic) to examine whether various aspects of quality of life are associated with depressive symptoms, hopelessness, or suicide ideations in forensic inpatients with substance use disorders. To distinguish individual factors from hospital-specific environmental factors, we considered both the patient and the hospital levels. Hospital characteristics were generated by aggregating the MQPL-forensic variables measured at the patient level. This approach allowed us to separate subjective (= patient level) from more objective (= hospital level) measures of quality of life.

## Methods

### Procedure

The study was funded by the Bavarian State Ministry of Family, Labour and Social Affairs, Germany, and approved by the ethics committee of the University of Ulm, Germany (application number: 176/17 and 174/17). It was performed in accordance with the Declaration of Helsinki.

Forensic psychiatric inpatients were included if they were 18 years or older and if, in the opinion of the professionals responsible for their treatment, they were able to give informed consent (i.e., if they had no acute symptoms of a mental disorder and no intellectual disability).

Patients were informed about the study objectives and about the fact that neither participation nor non-participation would have any advantages or disadvantages with respect to their treatment. After receiving this information, they could decide whether they were willing to participate in the study or not. Patients who agreed to participate gave written informed consent and received a sheet with contact details. Participants were able to withdraw their consent at any time. The study protocol included instructions on how to inform the patient and therapist if the assessments indicated an acute risk of self-harm. Patients received neither financial nor non-financial compensation for their participation. They completed the questionnaires in small groups in a separate room on the ward, and a research assistant was available to provide help.

### Sample

A total of 159 patients were recruited between February and August 2018 in 12 of the 14 forensic psychiatric hospitals in Bavaria, Germany. All the patients were detained according to Section 64 (substance use disorder) of the German Criminal Code. Seventeen patients could not be further evaluated because of incomplete data sets. The remaining sample consisted of 125 (88.0%) men and 17 (12.0%) women. The patients had a mean (SD) age of 33.15 (9.06) years (range 20–68 years) and had been treated for a mean (SD) of 12.55 (9.93) months (range 0-56 months). All 142 (100%) of the patients were diagnosed with a substance-related disorder (ICD-10: F10-F19), and some of them had a secondary diagnosis (multiple diagnoses were possible): 23 (16.2%) had a personality disorder (ICD-10: F60-F69); 8 (5.6%), depression (ICD-10: F30-F39); 3 (2.1%), schizophrenia (ICD-10: F20-F29); 4 (2.8%), anxiety or obsessive-compulsive disorder (ICD-10: F40-F48); and 1 (.7%), an eating disorder (ICD-10: F50). The index offenses, i.e., the offenses that led to the current admission, were as follows (multiple types of offense were possible): 78 patients were convicted because of violations of the Narcotics Act; 10, because of homicide; 13, because of robbery; 36, because of aggravated assault; 8, because of rape or sexual assault; 12, because of fraud; 25, because of theft; 5, because of arson; and 15, because of traffic offences. Nine patients did not provide precise information. A total of 17 (12.0%) patients had no educational qualifications; 76 (53.5%) had completed school to the end of grade 9 (“Hauptschulabschluss”), 38 (26.8%) had completed school to the end of grade 10 (“Realschulabschluss”), and 11 (7.7%) had graduated high school (“Abitur”).

### Materials

#### Assessment of Socio-Demographic, Clinical, and Legal Data

Patients were asked to provide the following information about themselves: Gender, age, highest school leaving qualification, duration of accommodation, diagnosis, legal basis for accommodation, index offence, and number of prior suicide attempts.

#### Measuring the Quality of Prison Life Adapted for Forensic Psychiatry (MQPL-Forensic)

In a former study, we translated the MQPL (12) questionnaire into German, adapted it to the living conditions of people in forensic psychiatry and supplemented it with questions on therapeutic support [see (25)]. The adapted version, the MQPL-forensic, consists of 64 items assigned to the following 11 subscales: entry into forensic psychiatry (4 items, Cronbach’s α = .599, example item “When I first came into this hospital I felt looked after”), relationship with fellow inmates (4 items, Cronbach’s α *r* = .678, example item “I have no difficulties with other patients in here”), relationship with caregivers (4 items, Cronbach’s α *r* = .843, example item “Relationships between staff and patients in this hospital are good”), relationship with therapists (7 items, Cronbach’s α *r* = .860, example item “I trust my therapist”), family contact (3 items, Cronbach’s α *r* = .588 example item “I am able to receive visits often enough in this hospital”), transparency of procedures and decisions (7 items, Cronbach’s α *r* = .810, example item “When important decisions are made about me, I am told how they came about”), fairness (5 items, Cronbach’s α *r* = .817, example item “Staff here treat patients fairly when applying the rules”), respect (6 items, Cronbach’s α *r* = .827, example item “I feel cared about most of the time in this hospital”), safety (6 items, Cronbach’s α *r* = .800, example item “This hospital is good at delivering personal safety”), quality of accommodation (11 items, Cronbach’s α *r* = .788, example item “I am given adequate opportunities to keep myself clean and decent”), and therapeutic options/personal development (7 items, Cronbach’s α *r* = .853, example item “I feel I have been encouraged to address my offending behavior”). The items were answered on a 5-point Likert scale from 0 (= I completely disagree) to 4 (= I completely agree). To evaluate the results, we calculated the mean scores of the subscales and the total scale. The higher the mean score, the more positively patients assessed individual aspects of their quality of life (= subscales) or their overall quality of life (= total score). The reliability of the adapted MQPL questionnaire was very good (Cronbach’s α of the total score: *r* = 0.951). The factor structure was checked by confirmatory factor analysis and was given (Chi²(1897) = 3442.143; *p* < .001; Bollen-Stine bootstrap *p* value = .008; root mean square error of approximation = .067; 90% confidence interval:.064 –.071) (25).

#### Beck Depression Inventory, Revised Version (BDI-II)

The revised version of the Beck Depression Inventory (BDI-II, (26), German version by (27) is a self-assessment tool for evaluating the severity of depressive symptoms. It consists of 21 categories, each consisting of four statements (example item: “I do not feel sad/I often feel sad/I always feel sad/I am so sad or unhappy that I can’t stand it.”). The statements are assigned scores from 0 to 3, and the total score is calculated by summing the scores. According to the manual, the German version of the BDI-II has good reliability and validity in hospital and non-hospital samples. The discrimination and internal consistency are also good (Cronbach’s α ≥ 0.89) (27).

#### Beck Hopelessness Scale (BHS)

The Beck Hopelessness Scale (BHS, (28); German version, [see (29)] assesses pessimism and negative expectations for the future. It comprises 20 items, each of which can be answered with “true” or “false” (example item: “My future seems dark to me”). Mean values are calculated for each item and the total score. For the internal consistency of the BHS, reliability coefficients between *r* = 0.72 and *r* = 0.97 are reported according to the Kuder-Richardson Formula 20 (29). The BHS adequately discriminates between people with and without suicide ideations (effect size by hedges = 0.62 – 3.43) and also adequately assesses the severity of suicide ideations (effect size by hedges = 1.19 – 1.97) (29).

#### Beck Scale for Suicide Ideation (BSS)

The Beck Scale for Suicide Ideation [BSS, (30)]; German version, [see (31)] comprises 21 items that assess active and passive suicide ideations, suicidal tendencies, past suicide attempts, and the severity of suicidality. The first five items serve as a screening for active and passive suicide ideations; statements 4 (“I have no desire to kill myself”) and 5 (“I would try to save my life if I find myself in a life-threatening situation”) of this screening are used as filter questions. If both statements are affirmed, the patient is categorized as a patient with suicide ideations (= above BSS cut-off). Reliability analysis of the BSS-screen revealed an excellent Cronbach’s α of *r = *.89 for internal consistency. Validity was demonstrated by correlating the BSS-screen score with other questionnaires measuring similar constructs [BHS (28): *r* = .36; Patient Health Questionnaire (32): *r* = .33] (31).

### Statistical Analyses

We analyzed data with IBM SPSS Statistics for Windows Version 25 (Armonk, NY: IBM Corp.). First, we calculated descriptive statistics (mean, standard deviations, absolute and relative frequencies) of all variables (MQPL-forensic, BDI-II, BHS, BSS). Next, we examined whether there were significant differences between the 12 forensic hospitals with regard to the assessed variables. Metric variables (MQPL-forensic, BDI-II, BHS) were analyzed by analyses of variance (ANOVAs) and frequencies (BSS), by Fisher’s exact test. Then, we used generalized estimating equation (GEE) models to evaluate the impact of quality of life on depression, hopelessness, and suicide ideations. GEE models allow for analyses of correlated outcomes, such as clustered data. In all analyses, hospital was added as a subject variable and defined the cluster membership of the patients. To estimate the covariance matrix, we accepted the default robust estimator (also called the Huber-White sandwich estimator). When predicting the dependent variables “mean BDI total score” and “mean BHS total score,” we specified the GEE model type linear (i.e., normal distribution and identity as link function). The following two regressor variables were calculated from the scores on the MQPL-forensic total scale and subscales: (1) the micro regressor, i.e., the deviation of an individual patient’s score from the mean value of the score at the patient’s hospital—in short the patient’s mean value was centered within the group (= hospital); (2) the macro regressor, i.e., the deviation of an individual hospital’s mean score from the mean score across all hospitals. The micro and macro regressors of each MQPL-forensic subscale and the total scale were added as independent variables in the GEE models. When predicting the dependent variables above or below the BSS cut-off score, we specified the GEE model type as a binary logistic model (i.e., binomial distribution and logit link function). The probabilities of the categories above the BSS cut-off score were modelled. As independent variables, we again included the micro and macro regressors for the MQPL-forensic total and subscale scores. The influence of possible covariates such as age, sex, duration of accommodation, and second diagnosis on the target variables was controlled.

## Results

**Table 1** shows the descriptive statistics of all variables. The mean MQPL-forensic total and subscale scores were between 2 and 3 and therefore tendentially in the positive range of the response scale (2 = I neither agree nor disagree; 3 = I agree). On the BDI-II, 20% of patients reported moderate to severe depression. Slightly fewer patients (9%) were classified as having moderate to severe hopelessness according to the guidelines of the BHS. According to the BSS, 6 patients (4%) had active or passive suicide ideations and 23 patients (17%) reported one or more past suicide attempts.

**Table 1:** Descriptive statistics for quality of life, depression, hopelessness, and suicide ideation in patients (N = 159) in 12 forensic psychiatric hospitals.

	Mean (SD)	Frequency
		*n* (%)
MQPL-forensic		
Entry into forensic psychiatry	2.20 (.70)	
Relationship with fellow inmates	2.22 (.73)	
Relationship with caregivers	2.54 (.69)	
Relationships with therapists	2.79 (.29)	
Family contact	2.33 (.73)	
Respect	2.55 (.64)	
Fairness	2.01 (.82)	
Transparency of procedures and decisions	2.19 (.71)	
Safety	2.55 (.63)	
Quality of accommodation	2.42 (.52)	
Therapeutic options/Personal development	2.99 (.55)	
Total score	2.48 (.44)	
BDI-II^1^	12.21 (10.35)	
No depression (0-8)		58 (44)
Minimal depression (9-13)		32 (24)
Mild depression (14-19)		15 (12)
Moderate depression (20-28)		17 (13)
Severe depression (29-63)		9 (7)
BHS^2^	3.90 (3.95)	
Minimal (0-3)		83 (63)
Mild (4-8)		37 (28)
Moderate (9-14))		8 (6)
Severe (15-20)		4 (3)
BSS		
Screen score	.35 (1.26)	
Total score	.78 (3.33)	
Affirm active and passive suicide ideations^3^		6 (4)
Deny active and passive suicide ideations^3^		136 (96)
No prior suicide attempt^4^		116 (84)
One suicide attempt^4^		9 (7)
Two or more suicide attempts^4^		14 (10)

^1^11 missing values, ^2^10 missing values ^3^In accordance with the manual, neither statement 4 nor statement 5 was marked with “0” (29), ^4^3 missing values.

MQPL-forensic, Measuring the Quality of Prison Life scale adapted for forensic psychiatry; BDI-II, Beck Depression Inventory; BHS, Beck Hopelessness Scale; BSS, Beck Scale for Suicide Ideation.

In the analyses of differences between the 12 hospitals by ANOVAs and Fisher’s exact tests, hospitals differed with regard to their patients’ mean BDI-II and BHS scores (see **Table 2**). In addition, they also differed significantly in the following MQPL-forensic subscales: entry into forensic psychiatry, respect, quality of accommodation, and therapeutic options/personal development.

**Table 2:** Results of tests analyzing differences between 12 forensic psychiatric hospitals in the quality of life, depression, hopelessness, and suicide ideation of their patients (N = 159).

	Statistics	Significance
BDI-II	*F*(11,130) = 2.012*	*p* = .032
BHS	*F*(11,130) = 2.492*	*p* = .007
BSS (Affirm/Deny suicide ideations)	Fisher‘s exact test = 10.904	*p* = .197
BSS (Prior suicides: yes/no)	Fisher‘s exact test = 15.393	*p* = .089
MQPL-forensic		
Entry into forensic psychiatry	*F*(11,130) = 4.274*	*p* < .001
Relationship with fellow inmates	*F*(11,130) = 1.841	*p* = .053
Relationship with caregivers	*F*(11,130) = 1.371	*p* = .194
Relationships with therapists	*F*(11,130) = .985	*p* = .464
Family contact	*F*(11,130) = 1.110	*p* = .358
Respect	*F*(11,130) = 2.043*	*p* = .029
Fairness	*F*(11,130) = .694	*p* = .742
Transparency of procedures and decisions	*F*(11,130) = .880	*p* = .562
Safety	*F*(11,130) = 1.634	*p* = .096
Quality of accommodation	*F*(11,130) = 2.293*	*p* = .013
Therapeutic options/personal development	*F*(11,130) = 2.171*	*p* = .020
Total score	*F*(11,130) = 1.034	*p* = .420

*p < .05.

MQPL-forensic, Measuring the Quality of Prison Life scale adapted for forensic psychiatry; BDI-II, Beck Depression Inventory; BHS, Beck Hopelessness Scale; BSS, Beck Scale for Suicide Ideation.

The GEE models predicting depressive symptoms (mean BDI-II total score) can be seen in **Table 3**. The MQPL-forensic total score and the scores of almost all the subscales were significant negative predictors on the patient level, i.e., a supportive welcome to the hospital; positive relationships with fellow inmates, caregivers and therapists; support in maintaining family contacts; respectful interactions; transparency of procedures and decisions; and a high feeling of safety were associated with significantly fewer depressive symptoms. For example, the BDI-II score was -.243 lower in patients who valued their welcome to the hospital more positively (+1 higher than the mean score in the patients). On the hospital level, a friendly welcome to the hospital, positive relationships with therapists, support in maintaining family contacts, respectful interactions, and transparency of procedure and decisions were significant negative predictors. For example, the patients’ BDI-II score was -.207 lower in the hospitals with a higher rating for entry into forensic psychiatry (+1 higher than the mean score in the hospitals). Regarding micro and macro regressors, only the MQPL-forensic subscale “relationships with therapists” significantly interacted with the BDI-II total score in that if a particular patient’s score for the relationship with the therapist was more positive (+1) than the mean score of all patients and if this patient was in a hospital with a score above the mean score for all hospitals (+1), the patient’s BDI-II score did not decrease by -.605 [= (-.142) + (-.463)], but only by -.218 [= (-.605) +.387].

**Table 3:** Results of the generalized estimating equation models: Micro (= patient level) and macro (= hospital level) regressors predicting the severity of depressive symptoms (BDI-II) for each subscale of the Measuring the Quality of Prison Life scale adapted for forensic psychiatry (MQPL-forensic) and the MQPL-forensic total score in a sample of patients (N = 159) at 12 forensic psychiatric hospitals.

	Patient level	Hospital level	Interaction
	*b*	*b*	*b*
MQPL-forensic subscales			
Entry into forensic psychiatry	-.243*	-.207*	.049
Relationship with fellow inmates	-.151*	.071	-.417
Relationship with caregivers	-.216*	-.290	.161
Relationships with therapists	-.142*	-.463*	.387*
Family contact	-,134*	-,321*	.148
Respect	-.207*	-.514*	.199
Fairness	-.069	.103	-.147
Transparency of procedures and decisions	-.220*	-.467*	.140
Safety	-.147*	-.099	.096
Quality of accommodation	-.037	-.354	.117
Therapeutic options/personal development	-.136	-.171	.643
MQPL-forensic total score	-.349*	-.643	.567

*p < .05; b = unstandardized regression coefficient; interaction = micro regressor x macro regressor.

**Table 4** displays the results of the GEE models predicting hopelessness (mean BHS total score). On the patient level, the MQPL-forensic total score and the scores on the subscales entry into forensic psychiatry, relationship with caregivers and therapists, family contact, respect, fairness, transparency of procedures and decisions, and therapeutic options/personal development were significant negative predictors of the BHS total score. On the hospital level, the subscales relationship with therapists, family contact, and respect were significant negative predictors. Furthermore, we observed a significant interaction: Patients who rated their relationships with fellow inmates more positively (+1 compared with the patients’ mean score) and who were in a hospital where this item was rated more positively (+1 compared with the hospitals’ mean score) were less depressed (-.303).

**Table 4:** Results of the generalized estimating equation models: Micro (= patient level) and macro (= hospital level) regressors predicting the severity of hopelessness (Beck Hopelessness Scale) for each subscale on the Measuring the Quality of Prison Life scale adapted for forensic psychiatry (MQPL-forensic) and the MQPL-forensic total score in a sample of patients (N = 159) at 12 forensic psychiatric hospitals.

	Patient level	Hospital level	Interaction
	*b*	*b*	*b*
MQPL-forensic subscales			
Entry into forensic psychiatry	-.067*	-.007	-.078
Relationship with fellow inmates	-.050	.101	-.303*
Relationship with caregivers	-.061*	-.093	.094
Relationships with therapists	-.067*	-.221*	.121
Family contact	-.058*	-.219*	.208*
Respect	-.086*	-.196*	.013
Fairness	-.043*	.048	.059
Transparency of procedures and decisions	-.066*	-.141	.088
Safety	-.072	-.036	-.134
Quality of accommodation	-.023	-.191	.136
Therapeutic options/personal development	-.120*	-.050	.018
MQPL-forensic total score	-.152*	-.205	.082

*p < .05; b = unstandardized regression coefficient; interaction = micro regressor x macro regressor

The results of the analyses predicting suicide ideation (BSS) can be seen in **Table 5**. The MQPL-forensic total score and the scores on five subscales were significant predictors on the patient level, i.e., a supportive welcome to the hospital, positive relationships with therapists, respectful interactions, transparency of procedures and decisions, and helpful therapeutic options were associated with decreased suicide ideations. On the hospital level, the macro regressors relationship with caregivers and relationship with therapists were significant predictors of lower BSS scores. Again, the interaction concerning the relationship with fellow inmates was significant: Patients who rated their relationships with fellow inmates more positively (+1 compared with the patients’ mean score) and who were in a hospital where this item was rated more positively (+1 compared with the hospitals’ mean score) reported significantly fewer suicide ideations (.005).

**Table 5:** Results of the generalized estimating equation models: Micro (= patient level) and macro (= hospital level) regressors predicting suicide ideations (Beck Scale for Suicide Ideation) for each subscale on the Measuring the Quality of Prison Life scale adapted for forensic psychiatry (MQPL-forensic) and the MQPL-forensic total score in a sample of patients (N = 159) at 12 forensic psychiatric hospitals.

	Patient level	Hospital level	Interaction
	Odds ratio	Odds ratio	Odds ratio
MQPL-forensic subscales			
Entry into forensic psychiatry	.261*	.523	7.232
Relationship with fellow inmates	.725	.064	.005*
Relationship with caregivers	.419	.192*	.242
Relationships with therapists	.209*	.006*	.622
Family contact	.552	1.457	2.490
Respect	.275*	.154	2.785
Fairness	.814	.395	1.894
Transparency of procedures and decisions	.193*	.057	1.080
Safety	.513	.813	1.692
Quality of accommodation	.633	2.466	2.675
Therapeutic options/personal development	.131*	.139	.252
MQPL-forensic total score	.110*	.037	1.159

*p < .05; interaction = micro regressor x macro regressor.

## Discussion

The aim of the present study was to determine whether there are relationships between various aspects of quality of life and depressive symptoms, hopelessness, or suicide ideations in forensic psychiatric patients with substance use disorders. The descriptive statistics showed that the surveyed patients had a high risk for depression and suicide ideations: About 20% of the patients had moderate to severe depressive symptoms, and 4% had active or passive thoughts of suicide. The high number of patients who had already attempted suicide (17%) indicates that these suicide ideations must be taken seriously.

The results of the present study show that the mental health of patients in forensic psychiatry is related to their quality of life: Almost all aspects of quality of life proved to be protective factors against depression and hopelessness. Suicide ideations occurred less frequently when patients experienced a friendly welcome to the hospital, positive relationships with therapists, respectful interactions, transparency of procedures and decisions, and supportive therapeutic options. The relationships described at the patient level are subjective because the study used a self-assessment tool for quality of life. To objectify quality of life, we therefore calculated mean values for each hospital. The results showed that the quality characteristics at the hospital level also influenced the patients’ mental well-being. Patients were less depressed, hopeless, or suicidal in hospitals in which the admission situation is positive, therapists have good relationships with patients, patients are supported in maintaining contact with their family members and treat each other respectfully, and procedures and decisions are transparent. In all three hospital-level analyses (BDI, BHD, and BSS), a good relationship with the therapist was a significant predictor of quality of life measured by the MQPL-forensic. Thus, the relationship with the therapist seems to be a very important variable. Patients who have a trusting relationship with their therapist may experience less strongly the loss of control associated with being in a forensic psychiatric hospital. Positive social relationships are repeatedly highlighted as protective factors for suicide (33, 34), and this appears to be the case also in forensic psychiatry. Our results clearly support the deprivation model because they show that inmates’ thoughts of suicide depend on environmental, hospital-level factors. Furthermore, our results are consistent with those reported by Liebling et al. (3) for prison inmates, i.e., that the more positive the dimensions respect, relationship, fairness, clarity, security, and family contact were evaluated, the lower the inmates’ psychological distress and the lower the prison suicide rate.

Finally, the quality of life in the 12 hospitals was rated very differently. We found differences with regard to the following aspects: entry into forensic psychiatry, respectful interactions, quality of accommodation, and therapeutic option. All these aspects can be changed by the head and staff of the hospitals. For example, the admission procedure can be precisely specified, e.g., the point when primary nurses and therapists are appointed and when they have the first conversation with the patient, and written ward rules can be created that state when patients are informed about the important activities throughout the day (meal times, etc.). Forensic-psychiatric hospitals should be aware of the significant importance of these dimensions for patients’ quality of life and adapt them if necessary to try to improve patients’ mental well-being.

### Limitations

The data were collected in an ex post facto design, so we can only report statistical associations and not direct causes or effects. It should be noted that we use the term predictor in the results section to describe the statistical function of the independent variable in a regression model and not a cause-effect relationship. Further, the described relationship between depression, hopelessness, suicide ideations, and quality of life in forensic psychiatry could also be mediated by other variables that were not investigated in the present study (e.g., neuroticism on the patient level). And finally, all variables were collected by using questionnaires, i.e., as self-disclosure. In surveys, respondents mainly want to give positive descriptions of themselves, which could influence the answers to stressful and sometimes shameful topics in particular, such as depression or suicidal behavior. It also should be noted that the present sample is very heterogeneous with regard to the second diagnosis of the participants, which can have an influence on the results. Furthermore, we did not have any information on the patients’ medication intake.

### Conclusion

The results show that interventions that create a positive environment for their patients, characterized by aspects of care rather than custodial, could reduce psychological stress among inmates. A positive and appreciative climate can be achieved through various measures: It is important that staff take care of the patients’ right from the start of admission, assign them a permanent contact person and familiarize them with the clinic’s procedures and rules. In everyday life, it becomes apparent that patients to whom decisions and measures are explained feel less hopeless and depressed. Psychologically disturbed offenders should be seen as a particularly vulnerable group whose institutional care requires increased elaborate organisational culture. It is important that the head and staff of forensic-psychiatric hospitals focus on all aspects of quality of life and provide the impetus for change because patients have little freedom of action to change their environment.

## Data Availability Statement

The datasets generated for this study are available on request to the corresponding author.

## Ethics Statement

The studies involving human participants were reviewed and approved by Ethikkommission der Universität Ulm. The patients/participants provided their written informed consent to participate in this study.

## Author Contributions

MD, SN, SO, and MB designed the study. MB collected the data. MB, ML, and SN analyzed the data. MB, JS, IF, and SN interpreted the data. MB wrote the initial draft of the manuscript. All authors had full access to all the data in the study and take responsibility for the integrity and accuracy of the data analysis. All authors contributed to read and approve the final version of the manuscript.

## Funding

The study was funded by the Bavarian State Ministry for Family, Labour and Social Affairs, Germany.

## Conflict of Interest

The authors declare that the research was conducted in the absence of any commercial or financial relationships that could be construed as a potential conflict of interest.

## References

[1] Statista (2019). Selbstmordrate in Deutschland nach Bundesländern und Geschlecht im Jahr 2016 (Suizide je 100.000 Einwohner). Hamburg, Germany: Statista Research Department Available at: https://de.statista.com/statistik/daten/studie/318394/umfrage/selbstmordrate-in-deutschland-nach-bundeslaendern-und-geschlecht/ (Accessed May 14, 2019).

[2] Voulgaris A Kose N Konrad N Opitz-Welke A Prison Suicide in Comparison to Suicide Events in Forensic Psychiatric Hospitals in Germany. Front Psychiatry (2018) 9:398. 10.3389/fpsyt.2018.00398 30210374 PMC6121193

[3] Liebling A Durie L Stiles A Tait S Revisiting prison suicide: the role of fairness and distress. In: Liebling A Maruna A, editors. The effects of imprisonment. London: Routledge (2005). p. 209–31.

[4] Dye MH Deprivation, importation, and prison suicide: Combined effects of institutional conditions and inmate composition. J Crim Justice (2010) 38(4):796–806. 10.1016/j.jcrimjus.2010.05.007

[5] Edgemon TG Clay-Warner J Inmate mental health and the pains of imprisonment. Soc Ment Health (2019) 9(1):33–50. 10.1177/2156869318785424

[6] Bani M Travagin G Monticelli M Valsecchi M Truisi E Zorzi F Pattern of self-injurious behavior and suicide attempts in Italian custodial inmates: A cluster analysis approach. Int J Law Psychiatry (2019) 64(3):1–7. 10.1016/j.ijlp.2018.12.008 31122619

[7] Yang S Kadouri A Révah-Lévy A Mulvey EP Falissard B Doing time: A qualitative study of long-term incarceration and the impact of mental illness. Int J Law Psychiatry (2009) 32:294–303. 10.1016/j.ijlp.2009.06.003 19619895 PMC2758061

[8] Black DW Gunter T Loveless P Allen J Sieleni B Antisocial personality disorder in incarcerated offenders: Psychiatric comorbidity and quality of life. Ann Gen Hosp Psychiatry (2010) 22(2):113–20. 20445838

[9] Schreiber J Culpepper L Suicidal ideation and behavior in adults. In: Solomon D, editor. Waltham, MA, USA: UpToDate Inc. (2010). Availabe at: https://www.uptodate.com/contents/suicidal-ideation-and-behavior-in-adults (Accessed May 14, 2019).

[10] Meltzer HY Suicidality in schizophrenia: a review of the evidence for risk factors and treatment options. Curr Psychiatry Rep (2019) 4:279–83. 10.1007/s11920-996-0047-6 12126596

[11] Radoschewski M Gesundheitsbezogene Lebensqualität – Konzepte und Maße. Bundesgesundheitsblatt – Gesundheitsforschung – Gesundheitsschutz (2000) 43(3):165–89. 10.1007/s001030050033

[12] Liebling A Hulley S Crewe B Conceptualising and measuring the quality of prison life. In: Gadd D Karstedt S Messner SF, editors. The Sage handbook of criminological research methods. London: Sage publications Ltd. (2011). p. 358–72. 10.4135/9781446268285.n24

[13] Bullinger M Kuhn J Leopold K Janetzky W Wietfeld R Lebensqualität als Zielkriterium in der Schizophrenietherapie. Fortschr Neuro Psychiatr (2019) 87(06):348–56. 10.1055/a-0646-3951 30541162

[14] Whoqol Group The World Health Organization quality of life assessment (WHOQOL): position paper from the World Health Organization. Soc Sci Med (1995) 41(10):1403–9. 10.1016/0277-9536(95)00112-K 8560308

[15] Tonkin M A review of questionnaire measures for assessing the social climate in prisons and forensic psychiatric hospitals. Int J Offender Ther Comp Criminol (2016) 60(12):1376–405. 10.1177/0306624X15578834 25850103

[16] Van Nieuwenhuizen C Schene AH Koeter MWJ Quality of life in forensic psychiatry: an unreclaimed territory? Int Rev Psychiatry (2002) 14:198–202. 10.1080/09540260220144993

[17] Liebling A Arnold H Measuring the quality of prison life. London: Home Office, Research, Development and Statistics Directorate (2002).

[18] Goldberg D General Health Questionnaire (GHQ-12). Windsor, UK: NFER-Nelson (1992).

[19] Müller JL Saimeh N Briken P Eucker S Hoffmann K Koller M Standards für die Behandlung im Maßregelvollzug nach §§ 63 und 64 StGB. Der Nervenarzt (2017) 88(1):1–29. 10.1007/s00115-017-0382-3 28776213

[20] Long C McLean A Boothby A Hollin C Factors associated with quality of life in a cohort of forensic psychiatric in‐patients. Br J Forensic Pract (2008) 10(1):4–11. 10.1108/14636646200800002

[21] Swinton M Oliver J Carlisle J Measuring quality of life in secure care: Comparison of mentally ill and personality disordered patients. Int J Sl Psychiatr (1999) 45(4):284–91. 10.1177/002076409904500407 10689612

[22] Vorstenbosch ECW Bouman YHA Braun PC Bulten EBH Psychometric properties of the forensic inpatient quality of life questionnaire: quality of life assessment for long-term forensic psychiatric care. Health Psychol Behav Med (2014) 2(1):335–48. 10.1080/21642850.2014.894890 PMC4346075 25750786

[23] Bouman YHA de Ruiter C Schene AH Quality of life of violent and sexual offenders in community-based forensic psychiatric treatment. J Forensic Psychi PS (2008) 19(4):484–501. 10.1080/14789940701877669

[24] Gerber GJ Prince PN Duffy S McDougall L Cooper J Dowler S Adjustment, integration, and quality of life among forensic patients receiving community outreach services. Int J Forensic Ment Health (2003) 2(2):129–36. 10.1080/14999013.2003.10471184

[25] Büsselmann M Streb J Dudeck M Development of a questionnaire on measuring the quality of life in forensic hospital. Unpublished manuscript (2019).

[26] Beck AT Steer RA Brown GK Beck Depression Inventory–II (BDI–II). San Antonio, TX Harcourt Assessment Inc. (1996). 10.1037/t00742-000

[27] Hautzinger M Keller F Kühner C Beck Depressions-Inventar (BDI-II). Revision. Frankfurt/Main: Harcourt Test Services (2006).

[28] Beck AT Steer RA Beck Hopelessness Scale (BHS). Bloomington, MN: Pearson (1993).

[29] Kliem S Brähler E Beck-Hoffnungslosigkeits-Skala (BHS). Deutsche Fassung. Manual. Frankfurt/Main: Pearson Assessment (2015).

[30] Beck AT Steer RA Beck Scale for Suicide Ideation (BSS). Bloomington, MN: Pearson (1993).

[31] Kliem S Brähler E Beck-Suizidgedanken-Skala (BSS) Deutsche Fassung. Manual. Frankfurt: Pearson Assessment (2015).

[32] Löwe B Kroenke K Gräfe K Detecting and monitoring depression with a 2-item questionnaire (PHQ 2). J Psychosom Res (2005) 58:163–71. 10.1016/j.jpsychores.2004.09.006 15820844

[33] Kleiman EM Liu RT Social support as a protective factor in suicide: Findings from two nationally representative samples. J Affect Disord (2013) 150(2):540–5. 10.1016/j.jad.2013.01.033 PMC3683363 23466401

[34] Miller AB Esposito-Smythers C Leichtweis RN Role of social support in adolescent suicidal ideation and suicide attempts. J Adolesc Health (2015) 56(3):286–92. 10.1016/j.jadohealth.2014.10.265 25561384

